# Bilateral lower limb dysfunction due to acute hematoma in the lumbar ligamentum flavum: A case report and literature review

**DOI:** 10.1097/MD.0000000000051056

**Published:** 2026-10-16

**Authors:** Bingyi Yang, Huoliang Zheng, Qizhu Chen, Hao Cai, Yong Wang, Muradil Mardan, Leisheng Jiang, Shengdan Jiang

**Affiliations:** a Department of Spine Surgery, Xinhua Hospital Affiliated to Shanghai Jiaotong University School of Medicine, Shanghai, China.

**Keywords:** acute pain, case report, disc herniation, hematoma, ligamentum flavum, lumbar spine

## Abstract

**Rationale:**

There have been several reports of lumbar yellow ligament hematomas leading to cauda equina and/or nerve root compression. However, based on clinical history and histological findings, most of these cases involve chronic hematomas. Acute lumbar yellow ligament hematomas causing nerve compression are relatively rare.

**Patient concerns:**

A 71-year-old male presented with an 8-day history of sudden-onset lower back pain without an identifiable trigger, which progressively worsened with new-onset pain and numbness in both lower extremities. Physical examination revealed muscle strength of the quadriceps femoris left IV/right V, tibialis anterior left IV/right IV, and extensor hallucis longus left V/right V.

**Diagnoses:**

Magnetic resonance imaging on the 8th day after symptom onset revealed severe stenosis of the L4/5 spinal canal and cauda equina compression caused by a posterior mass, which appeared hyperintense on T1-weighted images and hypointense on T2-weighted images with nodular and ring-like hyperintense signals. Histopathological and immunohistochemical examination confirmed ligamentum flavum hematoma with recent angiogenesis and hemorrhage in the degenerated yellow ligament.

**Interventions:**

Surgical exploration and resection of the extradural mass were performed on the 9th day after symptom onset. An endoscopic-assisted posterior spinal decompression, laminectomy, and spinal fusion with internal fixation at the L4/5 level were performed.

**Outcomes:**

The surgical treatment was successful, and the severe bilateral lower limb dysfunction improved immediately after surgery. Two weeks after surgery, at follow-up, muscle strength had been restored to grade 5 with no recurrence of symptoms.

**Lessons:**

Acute ligamentum flavum hematoma can occur without trauma and should be included in the differential diagnosis for sudden bilateral lower limb deficits. MRI is crucial for early recognition, and prompt surgical intervention can yield rapid, complete recovery, underscoring the importance of timely diagnosis and decompression to prevent permanent neurologic injury and inform evidence-based management.

**Key Points**

**Key findings**

1. Acute ligamentum flavum hematoma (LFH) at L4/5 caused bilateral lower‑limb dysfunction.
2. Early MRI captured stage‑dependent signal evolution.
3. Imaging indicated acute hematoma, corroborated by surgical histology.
4. Endothelial immunohistochemistry confirmed LFH.

**What is known and what is new?**

- Known
  - LFH is a rare cause of radiculopathy/cauda equina compression, typically at L4/5 in older adults with degeneration/instability.
  - Fragile neovessels in degenerated ligamentum flavum may rupture after minor trauma, repetitive motion, procedures, or spontaneously.
  - MRI varies by hematoma stage; LFH can mimic cysts/tumors. Histopathology is definitive; surgery is effective with deficits.
- New in this manuscript
  - Early contrast MRI showed a stage‑consistent pattern (T1 hyperintense, T2 hypointense with nodular/ring‑like foci), raising preoperative suspicion for LFH.
  - Comprehensive histology proved vascular‑origin hemorrhage within the ligament.
  - Endoscopic-‑assisted decompression with fusion achieved rapid recovery within 2 weeks.

**What is the implication, and what should change now?**

- Include LFH in the differential for acute/subacute lumbar deficits in older adults with degeneration.
- Obtain early contrast‑enhanced MRI and interpret signals by stage to prevent misdiagnosis.
- If deficits progress, expedite decompression; resection both treats and confirms diagnosis.
- Request targeted endothelial markers (CD31, CD34, ERG) when needed; consider fusion if instability is present.
- Recognize that peripheral enhancement may reflect inflammatory change, not neoplasm.

## 1. Introduction

The ligamentum flavum (LF) is the longest ligamentous structure in the human body, primarily functioning to maintain spinal stability and assist in spine flexion and extension.^[1]^ Various pathological changes can occur in the LF, including hypertrophy, calcification, ossification, and cyst formation. However, LF is rarely associated with hematoma, making it an uncommon cause of nerve root and spinal cord compression.^[2]^ The normal LF consists of elastic and collagenous fibers with relatively poor vascularization.^[3]^ When degenerative or hypertrophic changes occur in the LF, the proliferated vessels are prone to rupture and cause hemorrhage following minor trauma. The first cases of ligamentum flavum hematoma (LFH) were reported by Sweasey et al in 1992, who described 2 patients with spontaneous LFH causing spinal cord and nerve root compression, thereby establishing LFH as a distinct clinical entity.^[4]^ Most reported cases of LFH occur at the lumbar level due to the dynamic activities of the lumbar spine, where the ligament experiences transmitted extradural pressure, leading to easier rupture of small vessels. A review of reported LFH cases found that the majority involve male patients over the age of 60, although the precise etiology and sex predilection remain incompletely understood.^[5]^ Therefore, further research and more case reports will contribute to a deeper understanding of this disease. Here, we describe a rare case of a 71-year-old male patient who presented with gradually worsening lower back pain without apparent cause, accompanied by numbness and pain in both lower extremities. Based on the patient’s clinical symptoms and imaging findings, we diagnosed LFH, and the patient’s symptoms markedly improved immediately after surgery. Histopathological examination postoperatively confirmed the diagnosis of LFH.

## 2. Materials and methods

This study was approved by the Ethics Committee of Xinhua Hospital, Shanghai Jiaotong University School of Medicine. Written informed consent was obtained from the patient for publication of this case report and any accompanying images. All procedures performed in this study were in accordance with the ethical standards of the Declaration of Helsinki and its subsequent amendments.

## 3. Case presentation

A 71-year-old male presented with an 8-day history of sudden-onset lower back pain without an identifiable trigger, which progressively worsened with new-onset pain and numbness in both lower extremities. He initially did not take the symptoms seriously and underwent physiotherapy while taking oral medications aimed at improving blood circulation (specific agents and dosages not documented in the available records). However, these measures proved ineffective, and the patient’s lower back pain intensified, accompanied by pain and numbness in the left buttock, the outer and posterior aspects of the left lower leg, as well as the outer side of the right lower leg, the sole of the foot, and the toes. The patient also reported difficulties standing and walking. The specialist physical examination revealed that the strength of the extensor muscles of the knee (quadriceps femoris) was left IV/right V, the strength of the ankle dorsiflexors (tibialis anterior) was left IV/right IV, and the strength of the long extensor of the great toe (extensor hallucis longus) was left V/right V. The patient had no significant past medical history, except for a cholecystectomy performed 5 years ago and benign prostatic hyperplasia.

In the imaging examinations, the lumbar spine X-ray, both in the anteroposterior and lateral views, showed osteoporotic changes and osteophyte formation in the vertebral bodies of the lumbar spine (Fig. 1). The computed tomography scan without contrast enhancement revealed disc herniations at the L4/5 and L5/S1 levels, with a localized abnormal density within the spinal canal at the L4/5 level and a narrowed intervertebral space at L4/5 (Fig. 2). The enhanced magnetic resonance imaging (MRI) of the lumbar spine showed an abnormal signal behind the dural sac at the L4/5 level. The signal was hyperintense on T1-weighted images and hypointense on T2-weighted images, with heterogeneous signal patterns including nodular and annular hyperintensity. The extradural mass at L4/5 caused severe spinal stenosis and compression of the cauda equina (Fig. 3).

**Figure 1.**
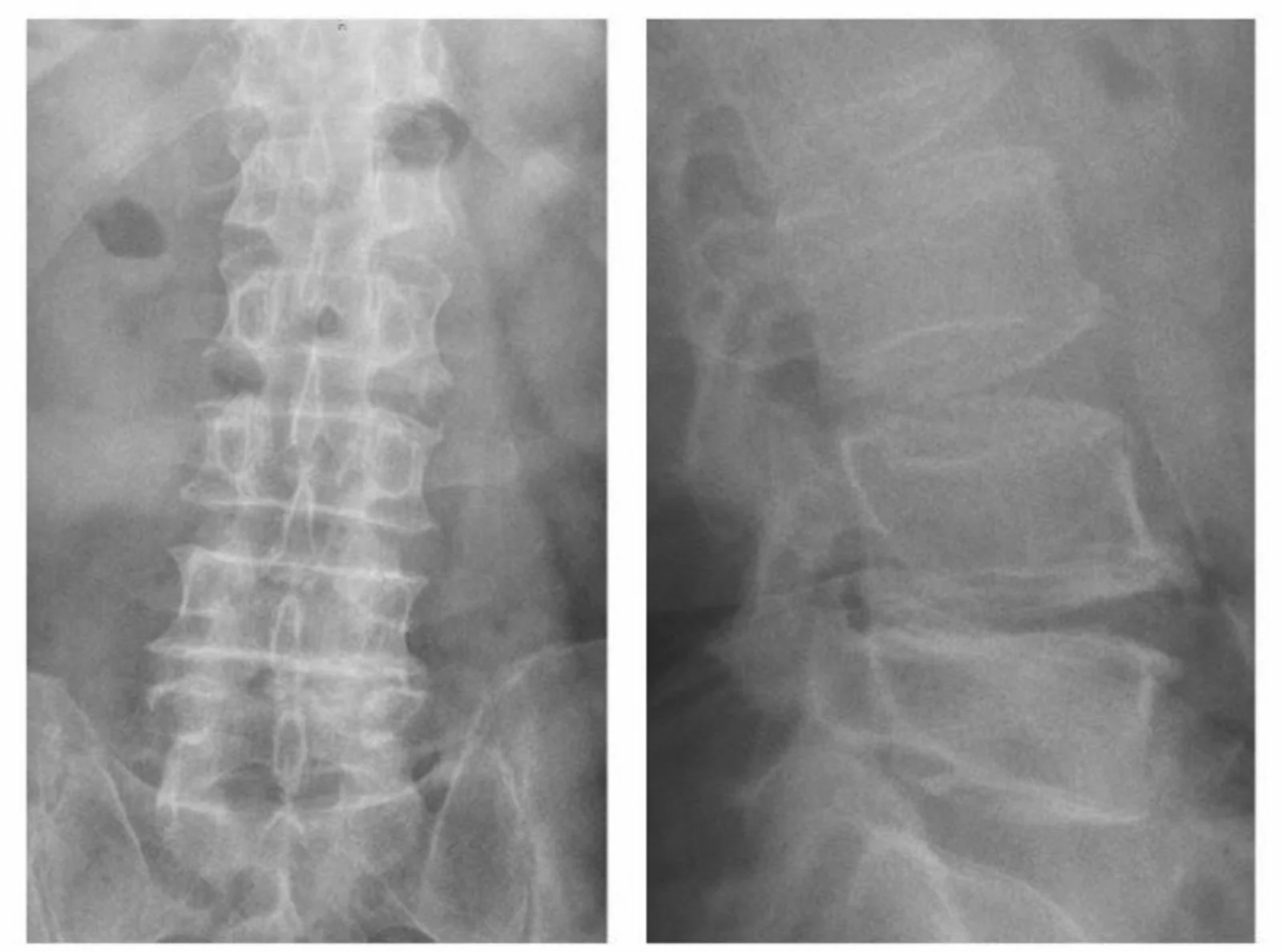
X-ray images show lumbar spine degenerative changes.

**Figure 2.**
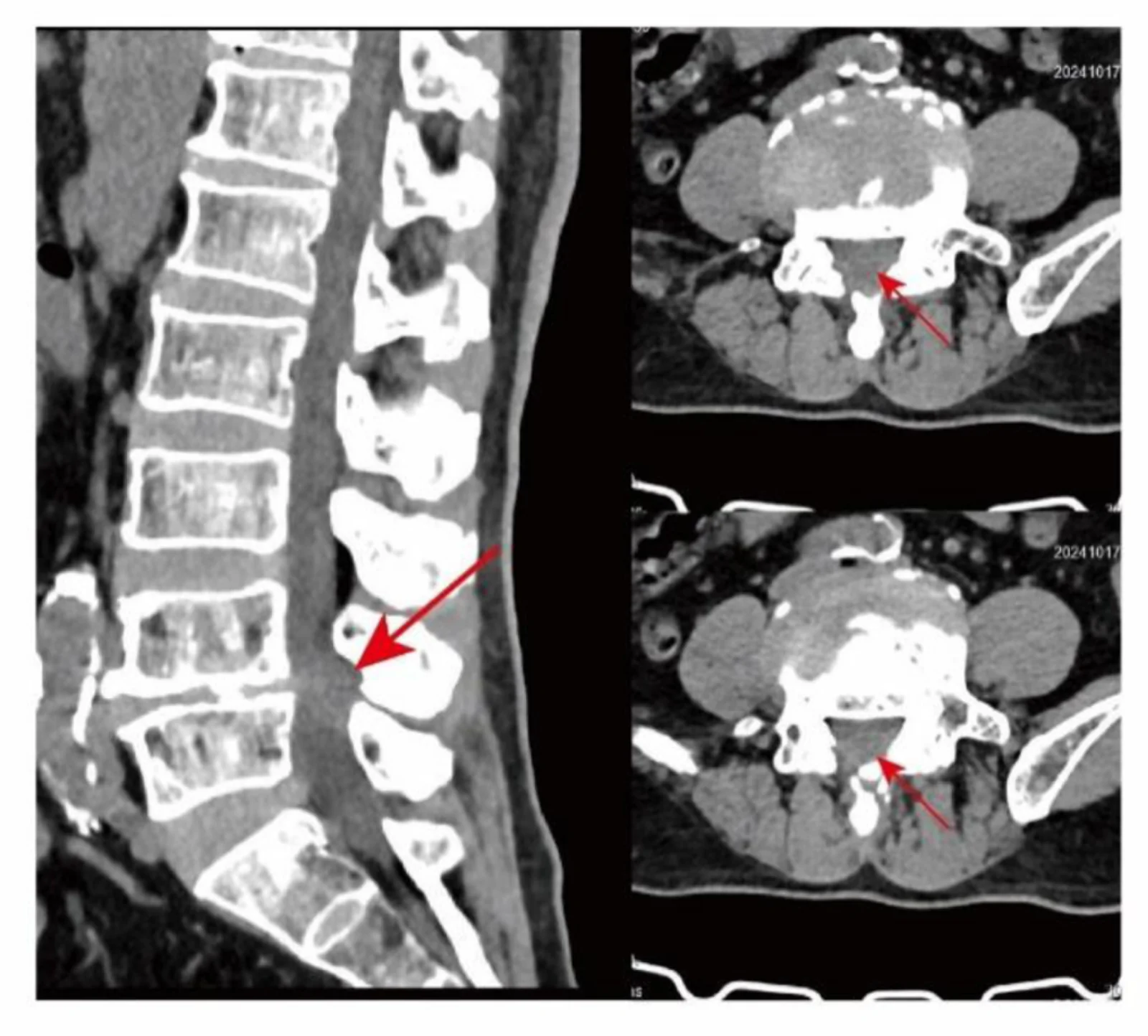
Preoperative CT imaging of the lumbar spine shows the lesion at the L4/5 segment on (left) sagittal and (right) axial views. CT = computed tomography.

**Figure 3.**
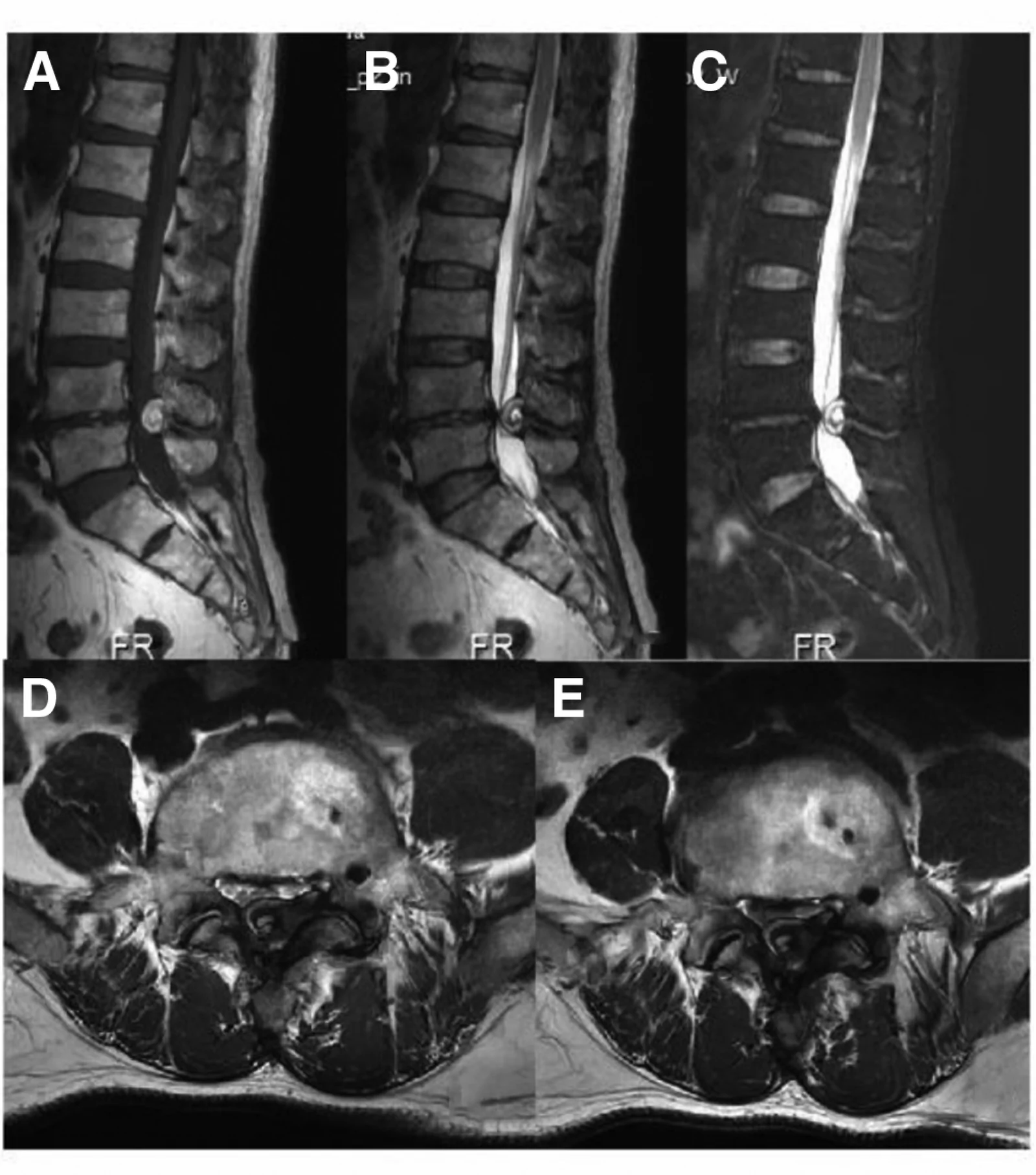
Preoperative enhanced MRI of the lumbar spine shows the lesion posterior to the dural sac at the L4/5 level, characterized by (A) high signal on sagittal T1-weighted MRI, (B) low signal on sagittal T2-weighted MRI with nodular and ring-like high signals, and heterogeneous signal intensity. (C) Sagittal fat-suppressed T2-weighted MRI, (D) axial T1-weighted, and (E) axial T2-weighted MRI shows a well-demarcated extradural lesion with a surrounding rim of hemosiderin and significant hypertrophy of the ligamentum flavum, resulting in severe spinal stenosis. MRI = magnetic resonance imaging.

Given the patient’s progressive symptoms and neurological deficits, the indications for surgery were clear. On the 9th day after symptom onset, the patient underwent an endoscopic-assisted posterior spinal decompression, laminectomy, and spinal fusion with internal fixation at the L4/5 level. During the surgery, under endoscopic assistance, the lower 2-thirds of the L4 lamina, the lower articular process (inferior facet), and the medial edge of the upper articular process (superior facet) of L5 were ground away to expose the LF. The hypertrophic and thickened LF was noted to be adherent to the dural sac. Following careful separation, dark red organized tissue was discovered within the LF. The hypertrophied LF and organized tissue were thoroughly excised to achieve complete decompression of the dural sac. Lastly, autologous bone grafting was employed for fusion, and pedicle screws were used for internal fixation (Fig. 4).

**Figure 4.**
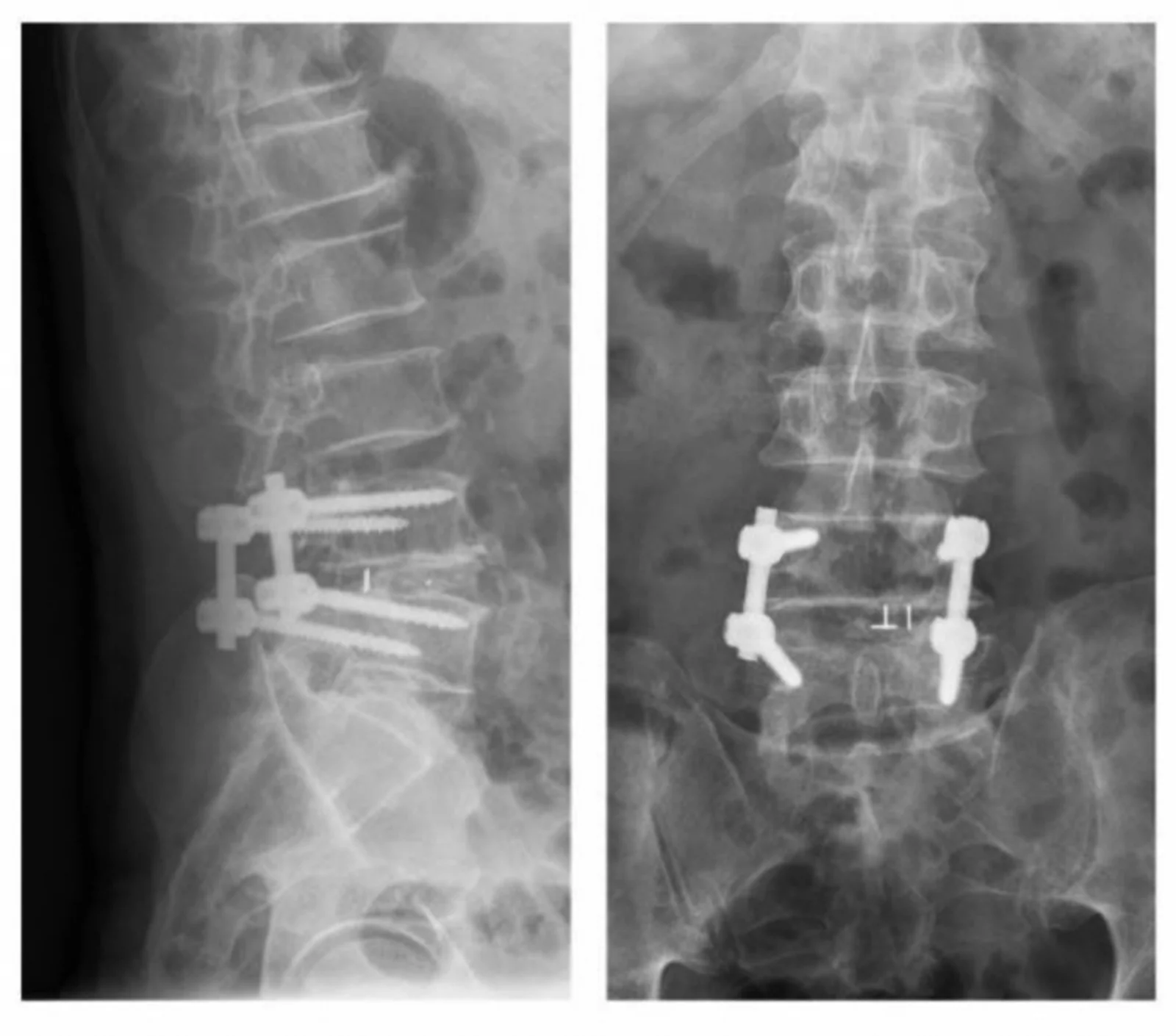
Postoperative X-ray images show the results of laminectomy and posterolateral interbody fusion at L4/5 with instrumentation, with (left) lateral view and (right) anteroposterior view.

The mass was completely removed, and histopathological examination of the surgical specimen showed the LF containing a small amount of degenerated fibrocartilage and fibrous wall-like tissue, accompanied by hemorrhage, local proliferation of small vessels, fibrous tissue proliferation, and hemosiderin deposition. Hematoma formation was confirmed by hematoxylin and eosin staining of the surgical specimen (Fig. 5). Immunohistochemical staining using cluster of differentiation 31 (CD31) and cluster of differentiation 34 (CD34) together with ERG, which are endothelial markers, yielded positive results (Fig. 5). CD31 is currently the most specific marker of endothelial differentiation, with no expression observed in almost all non-endothelial tissues. These histopathological results confirmed the diagnosis of LFH.

**Figure 5.**
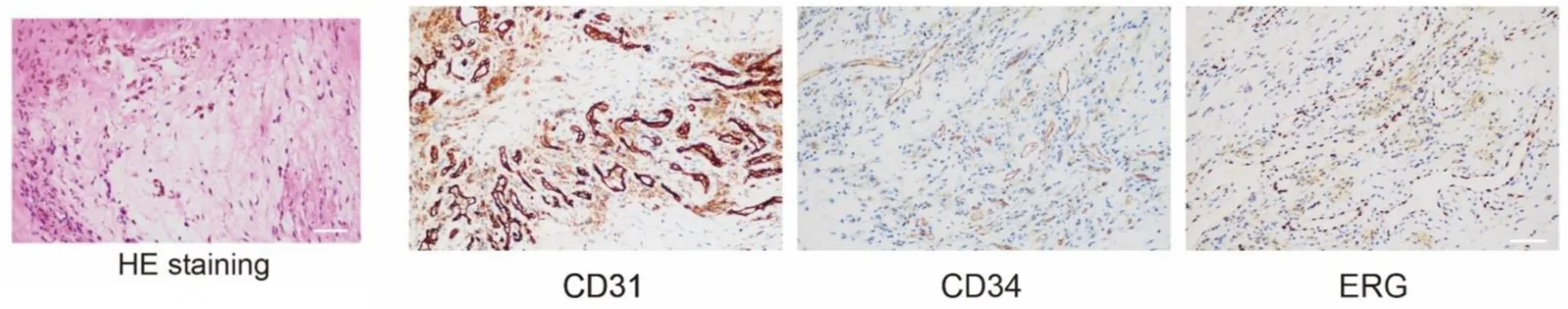
Microphotographs show the presence of hematoma. Hematoxylin and eosin and immunohistochemical staining of surgical specimen are positive for CD31, CD34 and ERG. CD31 = cluster of differentiation 31, CD34 = cluster of differentiation 34, ERG = ETS-1 related gene.

Surgical treatment proceeded smoothly, and the patient experienced gradual recovery of muscle strength in both lower extremities with improvement in numbness and pain postoperatively, after which they were discharged 3 days after surgery. Two weeks post-discharge, the patient returned for a scheduled follow-up visit and was reevaluated, showing full muscle strength recovery to grade 5 in both lower extremities, with no recurrence of symptoms. The patient reported full adherence to postoperative instructions and tolerated the procedure well, with no adverse events. The patient’s clinical course, diagnosis, and treatment are summarized in Table 1.

**Table 1:** Timeline of the patient’s clinical course, diagnosis, and treatment.

Time	Event	Details
Day 0	Symptom onset	A 71-year-old male experienced sudden-onset lower back pain without an identifiable trigger
Day 0–7	Symptom progression and conservative treatment	The lower back pain progressively worsened with new-onset pain and numbness in both lower extremities. Physiotherapy and medications to improve blood circulation were ineffective. Symptoms involved the left buttock, outer/posterior left lower leg, outer right lower leg, sole of the foot, and toes, with difficulty standing and walking. Physical examination on Day 8: quadriceps femoris L IV/R V, tibialis anterior L IV/R IV, extensor hallucis longus L V/R V
Day 8	Imaging diagnosis	X-ray: osteoporotic changes and osteophyte formation. CT: disc herniations at L4/5 and L5/S1; localized abnormal density in the spinal canal at L4/5 with narrowed intervertebral space. Enhanced MRI: extradural mass at L4/5, hyperintense on T1-weighted images and hypointense on T2-weighted images with nodular and ring-like hyperintense signals, causing severe spinal stenosis and cauda equina compression
Day 9	Surgical intervention	Endoscopic-assisted posterior spinal decompression, laminectomy, and spinal fusion with internal fixation at L4/5. Intraoperative findings: hypertrophic and thickened ligamentum flavum adherent to the dural sac, with dark red organized tissue within the ligamentum flavum. Complete decompression was achieved with autologous bone grafting and pedicle screw fixation
Day 9 (post-op)	Early postoperative recovery	Gradual recovery of muscle strength in both lower extremities with improvement in numbness and pain immediately after surgery
Day 12 (POD 3)	Hospital discharge	The patient was discharged 3 days after surgery with continued improvement
Week 2 (post-discharge)	Follow-up visit	Muscle strength fully recovered to grade 5 in both lower extremities. No recurrence of symptoms. Histopathology confirmed LFH: degenerated fibrocartilage, fibrous wall-like tissue, hemorrhage, small vessel proliferation, and hemosiderin deposition. Immunohistochemistry: CD31(+), CD34(+), ERG(+)

CD31 = cluster of differentiation 31, CD34 = cluster of differentiation 34, CT = computed tomography, ERG = ETS-1 related gene, LFH = ligamentum flavum hematoma, MRI = magnetic resonance imaging, POD = postoperative day.

Based on the above findings, we have summarized the following key results:

1. An unusual case of acute hematoma in the lumbar LF causing bilateral lower limb dysfunction is described.
2. In the early stages after the onset of symptoms, MRI examinations clearly demonstrated the intensity changes of the lesion.
3. These MRI findings indicated acute hematoma, a conclusion supported by the histologic findings of the LF resected at surgery.
4. Specific immunohistochemical results confirmed the diagnosis of LFH.

## 4. Discussion

This case report describes a 71-year-old male who presented with sudden-onset low back pain without an identifiable trigger, which progressively worsened over 8 days with new-onset pain, numbness, and weakness in both lower extremities. Contrast-enhanced MRI performed on the 8th day after symptom onset revealed a posterior extradural mass at the L4/5 level causing severe spinal canal stenosis and cauda equina compression, with signal characteristics suggestive of an acute hemorrhage. On the 9th day after symptom onset, the patient underwent endoscopic-assisted posterior spinal decompression, laminectomy, and fusion with internal fixation at the L4/5 level, during which dark red organized tissue was identified within the hypertrophic LF. Histopathological and immunohistochemical examination confirmed an acute LFH with recent angiogenesis and hemorrhage in the degenerated ligament. The patient’s bilateral lower limb dysfunction improved immediately after surgery, and at the 2-week follow-up his muscle strength had recovered to grade 5 with no recurrence of symptoms.

The LF is composed mainly of elastic fibers (80%) and collagen (20%) and has poor vascularization, with only a few small vessels passing through it in young, healthy individuals; thus, intraligamentous bleeding is extremely rare.^[6]^ The normal LF exhibits high resistance to injury; however, ligamentous degeneration is a well-recognized predisposing factor for LFH: in the degenerated LF, elastic fibers become dispersed, collagen content increases, and fibrous tissue granulation, lymphocytic infiltration, and proliferation of small capillaries occur. These neovascularization changes render the degenerated ligament susceptible to hemorrhage from physical trauma or mechanical stress, such as back stretching or repetitive microtrauma. In their report on hemorrhagic synovial cysts – a pathology that shares anatomical proximity and a similar degenerative milieu with LFH – Xu et al found that 8 of 30 reviewed patients had a documented history of physical trauma, suggesting that initial trauma and subsequent microtrauma may contribute to the pathogenesis of hemorrhage in juxta-facet cystic lesions^[7]^; extrapolating from this mechanism, microtrauma from daily activities or sports may similarly precipitate hemorrhage within the degenerated LF. Indeed, the currently widely accepted mechanism of LFH involves degeneration and hypertrophy of the LF with hemorrhage from fragile, neovascularized small vessels.^[8]^ Previous studies have noted the association between spondylolisthesis, facet joint disease, degenerative disc disease, and LFH, suggesting that degenerative changes and spinal instability contribute to the pathogenesis of LFH.^[9]^ A mechanistic study by Hur et al demonstrated that mechanical stress caused by spinal instability promotes angiogenesis in the LF, which serves as a critical link coupling mechanical stress to LF hypertrophy and may also predispose the ligament to hemorrhage.^[9]^ In addition, percutaneous procedures such as lumbar puncture or epidural interventions and elevated intra-abdominal pressure can cause vessel rupture, and hematomas may also occur in an idiopathic form without any apparent cause.^[10]^ In the present case, the LFH arose within a degenerated LF adjacent to the narrowed L4/5 intervertebral space.

The clinical manifestations of LFH are similar to those of more common causes of neural compression, such as intervertebral disc herniation. When LFH occurs in the lumbar region, it often presents with low back pain or radicular leg pain accompanied by neurological deficits, as seen in this patient.^[11,12]^ LFH typically occurs acutely or subacutely, with chronic LFH being rare, making it an uncommon but severe cause of neural compression.^[5]^

MRI is the optimal imaging modality for diagnosing LFH.^[12]^ However, given the rarity of the condition, distinguishing LFH from other posterolateral extradural lesions, such as juxta-facet cysts and intraspinal tumors, can be challenging.^[6,12]^ Synovial cysts and ganglion cysts are difficult to differentiate from one another and are collectively referred to as facet joint cysts. They may originate from the facet joints, LF, or posterior longitudinal ligament, are mostly found in degenerative lumbar spines, with over 50% occurring at the L4/5 level, and most commonly present with radicular pain.^[11,13]^ LF cysts, which arise from the inner surface of the ligament itself, are anatomically the closest cystic mimic of LFH. Importantly, hemorrhagic complications of facet joint cysts or LF cysts are extremely rare, with a reported probability of <10%^[11]^; therefore, when a posterolateral extradural lesion at a degenerative lumbar segment exhibits imaging features of acute hemorrhage, LFH should be strongly suspected in the differential diagnosis. This reasoning is further supported by the segmental distribution of LFH. In a review of the literature, Takahashi et al summarized 58 cases of LFH, of which 57 occurred in a single interlaminar segment – L4/5 (25 cases), L3/4 (15 cases), L2/3 (9 cases), and L5/S1 (4 cases)^[14]^ – indicating that the segments prone to LFH coincide with those affected by lumbar degenerative diseases, as observed in the present case. Morphologically, the rounded intraspinal appearance of LFH on MRI may be mistaken for an intraspinal tumor. Unlike spinal tumors, however, hemorrhagic enhancement is very rare in LFH, although mild to moderate peripheral enhancement may occur. Sahri and Tlemcani recently reported a case of LFH in which imaging showed significant enhancement, leading to a preoperative suspicion of a spinal tumor.^[15]^ We hypothesize that such enhancement reflects inflammatory reactions around the hematoma that increase vascular permeability and allow contrast agent leakage. Contrast-enhanced MRI may therefore be helpful in the differential diagnosis.

Because the MRI appearance of a hematoma varies with the time elapsed since bleeding, careful interpretation of signal characteristics in relation to the clinical time course is essential.^[12]^ Typically, within the first 3 days after bleeding, the conversion of oxyhemoglobin to deoxyhemoglobin results in a low signal on T2-weighted images, while the lesion remains isointense on T1-weighted images. During the early subacute phase (3–7 days), methemoglobin causes a high signal on T1-weighted images. Weeks later, hemolysis of red blood cells leads to extracellular accumulation of methemoglobin, resulting in iso- to hyperintense signals on both T1- and T2-weighted images.^[16]^ In the present case, contrast-enhanced MRI performed on the 8th day after symptom onset showed a mass that was hyperintense on T1-weighted images and hypointense on T2-weighted images, accompanied by nodular and ring-like hyperintense signals, indicating that the hematoma was in the acute-to-subacute transitional phase. These imaging findings were consistent with the clinical course and supported the preoperative suspicion of LFH.

For patients with severe pain and neurological deficits due to LFH, the first-line treatment is surgical excision, and the outcomes are generally good.^[12,14]^ In this case, given the patient’s severe pain and progressive neurological symptoms caused by the lesion, surgery was the optimal choice. Therefore, we opted for a posterior lumbar decompression, bone grafting, and internal fixation procedure. The patient had a good postoperative prognosis. Histopathological examination of the surgical specimen revealed a small amount of degenerated fibrocartilage and fibrous wall-like tissue with hemorrhage, localized small blood vessel proliferation, fibrous tissue hyperplasia, and hemosiderin deposition, confirming the diagnosis of LFH. In this context, we believe that surgery can serve as both a definitive therapeutic intervention and a reliable means to obtain an accurate histopathological diagnosis.

## 5. Conclusions

We report a rare case of acute LFH that occurred spontaneously, without an apparent trigger. Given the rarity of this condition, an accurate diagnosis requires a comprehensive evaluation of clinical symptoms, imaging results, and histopathological findings. The attending physician’s rich clinical experience is also crucial during this process. Surgery was the only reliable treatment option in this case, and the patient achieved an excellent outcome postoperatively.

## Acknowledgments

We would like to thank all the authors for their contributions, the patient for their cooperation, and the sponsors for their generous support.

## Author contributions

**Conceptualization:** Shengdan Jiang, Bingyi Yang, Huoliang Zheng, Qizhu Chen, Hao Cai, Yong Wang, Muradil Mardan, Leisheng Jiang.

**Data curation:** Shengdan Jiang, Bingyi Yang.

**Funding acquisition:** Shengdan Jiang, Huoliang Zheng.

**Investigation:** Bingyi Yang, Qizhu Chen, Muradil Mardan.

**Methodology:** Qizhu Chen, Hao Cai, Yong Wang.

**Project administration:** Bingyi Yang, Shengdan Jiang.

**Software:** Muradil Mardan.

**Supervision:** Bingyi Yang, Shengdan Jiang, Leisheng Jiang.

**Writing – original draft:** Bingyi Yang, Huoliang Zheng, Qizhu Chen, Hao Cai, Yong Wang, Muradil Mardan.

**Writing – review & editing:** Shengdan Jiang, Bingyi Yang, Huoliang Zheng, Leisheng Jiang.
